# Analysis of coniferous tree growth gradients in relation to regional pollution and climate change in the Miyun Reservoir Basin, China

**DOI:** 10.1007/s11356-023-26295-9

**Published:** 2023-03-10

**Authors:** Li Fu, Yan Xu, Dan Zhao, Bingfang Wu, Zhihong Xu

**Affiliations:** 1 State Key Laboratory of Remote Sensing Science, Aerospace Information Research Institute, Chinese Academy of Sciences, Beijing, 100101 China; 2 Centre for Planetary Health and Food Security and School of Environment and Science, Griffith University, Nathan, QLD 4111 Australia; 3 University of Chinese Academy of Sciences, Beijing, 100049 China

**Keywords:** Tree ring, Tree growth, Climate change, Pollution

## Abstract

Forests play a crucial role in regulating regional climate and mitigating local air pollution, but little is known about their responding to such changes. This study aimed to examine the potential responses of *Pinus tabuliformis*, the major coniferous tree species in the Miyun Reservoir Basin (MRB), along an air pollution gradient in Beijing. Tree rings were collected along a transect, and ring width (basal area increment, BAI) and chemical characteristics were determined and related to long-term climatic and environmental records. The results showed that *Pinus tabuliformis* showed an overall increase in intrinsic water-use efficiency (*iWUE*) at all sites, but the relationships between *iWUE* and BAI differed among the sites. The contribution of atmospheric CO_2_ concentration (*c*_*a*_) to tree growth was significant at the remote sites (> 90%). The study found that air pollution at these sites might have caused further stomatal closure, as evidenced by the higher δ^13^C levels (0.5 to 1‰ higher) during heavy pollution periods. The analysis of tree ring δ^15^N also revealed the potential of using δ^15^N to fingerprint major nitrogen (N) deposition, as shown in the increasing tree ring δ^15^N, and major nitrogen losses due to denitrification and leaching, as shown in the higher δ^15^N in tree rings during heavy rainfall events. Overall, the gradient analysis indicated the contributions of increasing *c*_*a*_, increasing water deficit and elevated air pollution to tree growth and forest development. The different BAI trajectories suggested that *Pinus tabuliformis* has the ability to adapt to the harsh environment in the MRB.

## Introduction

Forests cover approximately 31% of the Earth’s land surface (FAO and UNEP 2020) and provide numerous benefits and services to society, including water conservation, habitat, recreation, and regulation of local to global climates (Prevedello et al. 2019; Hu et al. 2020).

Studies have shown that climate change over the past two centuries has significantly impacted forest development worldwide (Seidl et al. 2017). For example, the increasing atmospheric CO_2_ concentration (*c*_*a*_) has both positive and negative effects on plant growth, depending on the type of plant and stand properties of the forest ecosystem (Cao and Woodward 1998; Beerling and Mayle 2006; Xu et al. 2014; Miller et al. 2016; Hisano et al. 2019). Changes in temperature, rainfall, and other factors have also been extensively reported to impact forest development (Pastor and Post 1988; Krankina et al. 2005; Baccini et al. 2012; Seidl et al. 2017).

In addition to climate change, forests are exposed to environmental pollution (Špulák and Souček 2010; Sun et al. 2010; Matyssek et al. 2012; Nowak et al. 2014; Succarie et al. 2020). The effects of pollution on forest development can be categorized into three classes (Smith 1990), with class I being low dosage with forests acting as a sink of air contaminants, class II being intermediate dosage with specific tree species facing adverse effects, and class III being high dosage resulting in morbidity or mortality of some tree species (Lorenz et al. 2010). Studies have shown that air pollution, particularly high concentrations of gaseous pollutants, is a major contributor to forest decline in some regions (Kandler and Innes 1995). Deposition of pollutants such as sulfur (S) and nitrogen (N) can significantly alter plant nutrition and soil chemistry and cause forest decline (Schulze 1989). It is essential to study the reactions and responses of forests, especially urban forests and forests surrounding megacities, which are projected to experience serious pollution issues (De Fries and Pandey 2010, Zhang et al. 2017).

In Beijing, forest ecosystems play a crucial role in protecting the soil and water and ensuring drinking water in water source areas. Additionally, forests help mitigate local air pollution by intercepting large amounts of particulate pollutants that are blown in from northwest provinces (Zhang et al. 2010, 2016). Efforts to create the current forest belt around Beijing began in the early 1960s, and the sheltering effect has been apparent. However, the impact of pollutants on tree growth and forest development, especially after the implementation of the Reform and Open-up Policy in the late 1970s that led to rapid population growth and economic development resulting in increased air pollution, is not well understood.

This study aimed to explore tree growth dynamics in response to changing climatic and environmental conditions in forests surrounding Beijing using *P. tabuliformis* (also known as Chinese pine) in the Miyun Reservoir Basin (MRB) as the target species. The specific objectives of the study were to (1) understand the long-term climatic and pollution conditions within the MRB based on available meteorological records and pollution observations, (2) determine tree growth profiles along a transect across the MRB using tree ring parameters, and (3) identify the contributions of changed climate conditions and pollution levels to tree growth during the studied period. The results are expected to provide insights into the impacts of air pollution on regional forest development and the ability of *P. tabuliformis* to cope with both global climate change and local pollution in Beijing, China.

## Materials and methods

### Study area

**Fig. 1:**
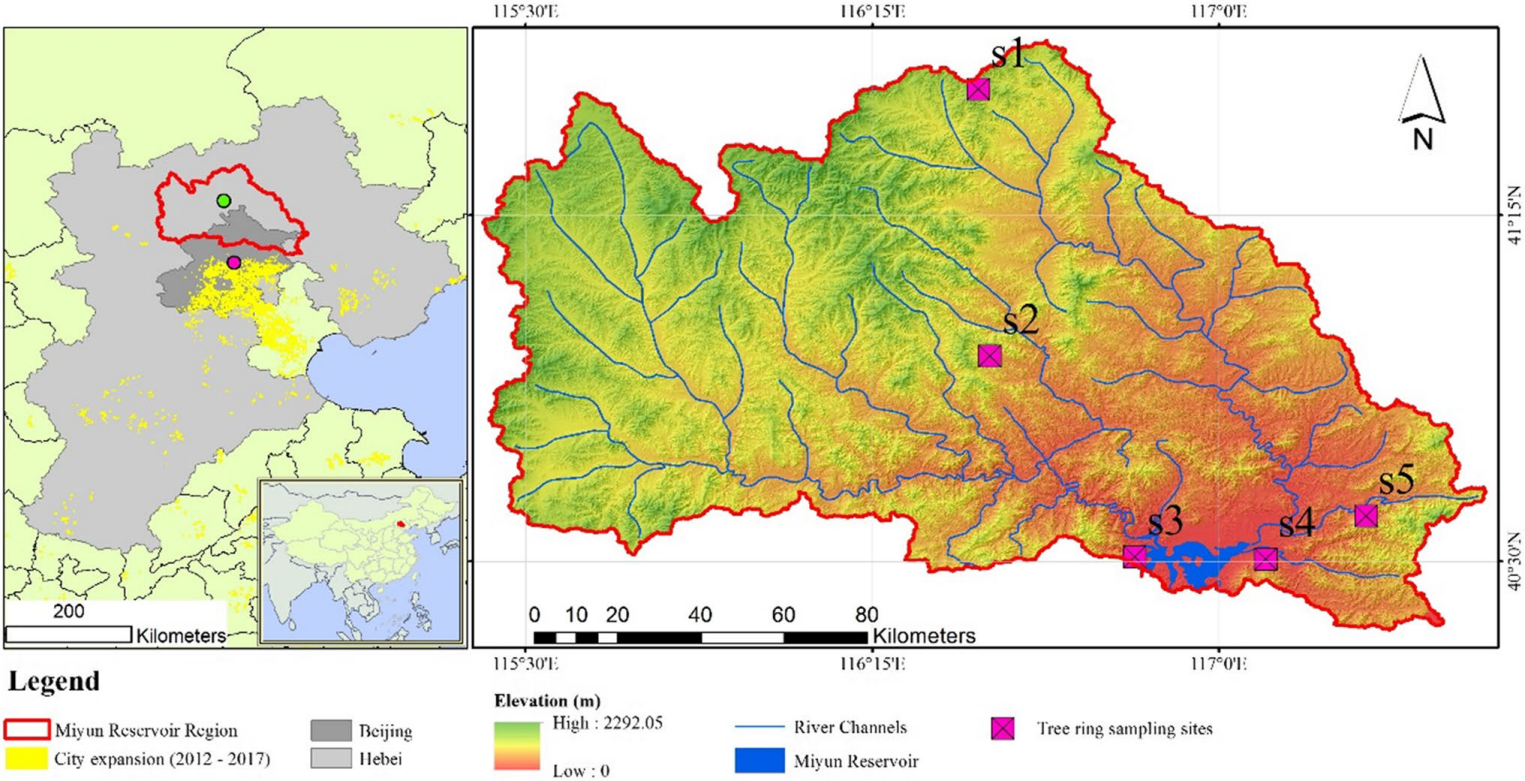
Study area and location of the sampling sites for gradient analysis. The green and purple spots (left panel) indicate the approximate locations of the Fengning and Miyun meteorological Stations, respectively

This study was carried out in the Miyun Reservoir Basin (MRB). The MRB is the major drinking water source to Beijing and is located approximately 180 km from the city center. The MRB (Fig. 1) is jointly administered by Beijing and Hubei Province, occupying approximately 15,500 km^2^. To the north and west, the basin is dominated by mountainous landscapes, with elevations up to approximately 2300 m above sea level, whereas to the southeast part of the basin, the terrain is relatively flat with a distribution of small hills but is mainly occupied by river plains.

Both climatic and environmental conditions within the MRB show spatial variations in response to topographic changes. The MRB is in a warm temperate zone and presents a typical continental monsoon climate with four distinct seasons. The annual average rainfall is approximately 669 mm, and its spatial distribution generally decreases from the southeast to northwest regions. The average temperature in the mountainous areas is significantly lower than that in the south-eastern part of the basin. The environmental condition, represented by the concentration of pollutants, also exhibits a certain gradient due to the characteristics of regional winds. The prevailing wind within the MRB is from the north and northwest, and the wind speed decreases due to the shelter effect of the mountains and forests. Since the concentrations of air pollutants generally decrease with increasing wind speed (He et al. 2001), particulate pollutants blown from the Gobi Desert are likely to accumulate on the south-eastern side of the MRB. Additionally, pollutants generated in Beijing are likely to show a decreasing temporal trend along the “south to north” direction due to wind resistance.

Considering the changes in climatic and pollution variations, a gradient analysis was selected to understand the trend of varied climatic conditions and pollutant concentrations and their impacts on local tree growth and forest development. As illustrated in Fig. 1, five sites (S1 to S5) along a transect were selected as the sampling sites. Generally, according to the previous descriptions, S1 and S5 represent the sites with the lowest impacts from pollution, while S2, S3, and S4 have experienced moderate to intensive impacts of pollution.

### Site descriptions

The five sites were selected according to the following criteria:

1. Mainly consist of *P. tabuliformis*;
2. Located away from major roads and counties to minimize additional impacts including pollutants from transportation (more N deposition) and human intervention (irrigation, fertilization, etc.);
3. Located away from water sources, including reservoirs, ponds, and rivers to avoid significant contributions of water.

At each site, physical properties (elevation, slope, aspect) were determined in situ*.* Surface soil samples (0–10 cm) were also collected to analyze their basic chemical properties. As demonstrated in Table 1, the five sites are located at different elevation levels, ranging from approximately 200 to 1300 m above sea level. Soils at the sites are quite thin and dominated by brown forest soil. All sites have slightly acidic soil (pH ranging from 6.5 to 6.9) and share similar nutrient conditions (comparable total N, total C, total phosphorus, and available phosphorus levels). Four to nine trees of *P. tabuliformis*, depending on the scale of the site and the density of trees, with varied ages at each site, were selected for tree ring sampling.

**Table 1:** Site properties, general soil characteristics, and tree properties at the five sampled sites

Site	Site properties			Soil properties					Trees	
	Elevation (m a.s.l)	Slope ( º)	Aspect (degree)	TN (%)	TC (%)	TP (mg kg^−1^)	AP (mg kg^−1^)	pH	Number of trees sampled	Tree age (years)
S1	1301	14	310	0.25	2.91	454	4.75	6.47	5	27–31
S2	798	13	120	0.12	1.54	378	4.44	6.64	4	24–42
S3	203	15	-	0.16	2.05	685	3.39	6.88	9	24–42
S4	358	11	245	0.15	1.71	467	4.64	6.86	5	30–49
S5	1152	19	80	0.26	2.98	340	3.42	6.91	4	26–31

TN, TC, TP, and AP represent total nitrogen, total carbon, total phosphorus, and available phosphorus

### Tree ring-based parameters

#### Sampling and preparation

Tree ring increment samples were collected according to the methods described in the previous studies (Xu et al. 2014). In brief, increment borers were used to extract two cores of two perpendicular directions at breast height for each selected tree. The samples were stored and transported to the tree ring laboratory at Griffith University. All the cores were then dried, sanded, and polished for dating and ring width measurement. Dating (and cross dating) and width measurements were performed with the LINTAB digital positioning table (Rinntech, Heidelberg, Germany).

#### Stable isotope analysis

This study adopted the whole wood sample method to analyze tree ring carbon (C) isotope composition (δ^13^C) and nitrogen (N) isotope composition (δ^15^N) (Loader et al. 2003). The two increment cores of each tree were pooled to generate a single sample, while rings from every 3 years period were pooled to minimize short-term noise. Wood samples representing 3-year increments for each tree were carefully split out in sequence from the pith to bark of the ring with a sharp blade. The samples were pooled every 3 years for two main reasons: first, to reduce the interannual noise, and second, to ensure a sufficient volume of samples for isotope analysis, as specified below (Fu et al. 2020). The pooled samples were oven dried at 60 °C until the weights of individual samples did not change. The samples were then ground to fine powder. Approximately 10 mg of sample was weighed with a fine balance and sealed in a tin capsule for stable isotope analysis (Xu et al. 2014). Outputs of the runs were the isotope ratio values expressed as relative deviation from the international standards.

#### Tree growth and water-use efficiency

Instead of using the measured ring width, which might biologically decline in mature trees, to present annual growth, this study used the basal area increment (BAI). The BAI is capable of providing consistent records of tree growth and thus has been accepted for long-term studies. The BAI can be calculated through the following equation:

1 $$\mathrm{BAI}=\uppi \times ({R}_{n}^{2}-{R}_{n-1}^{2})$$

where *R* is the tree radius at breast height, and *n* stands for the year when the ring was formed.

The intrinsic water-use efficiency (*iWUE*), defined as the rate of CO_2_ assimilation (*A*) divided by the stomatal conductance for water vapor (*g*_*s*_), was estimated according to the following equation:

2 $$iWUE={~}^{A}\!\left/ \!{~}_{{g}_{s}}\right.={~}^{({c}_{a}-{c}_{i})}\!\left/ \!{~}_{1.6}\right.$$

where *c*_*a*_ and *c*_*i*_ are the atmospheric CO_2_ concentration and intercellular CO_2_ concentrations, respectively. Equations for quantifying *c*_*a*_ and *c*_*i*_ are detailed in Fu et al. (2020).

### Reconstruction of long-term climatic and pollution profiles

#### Climatic variables

**Fig. 2:**
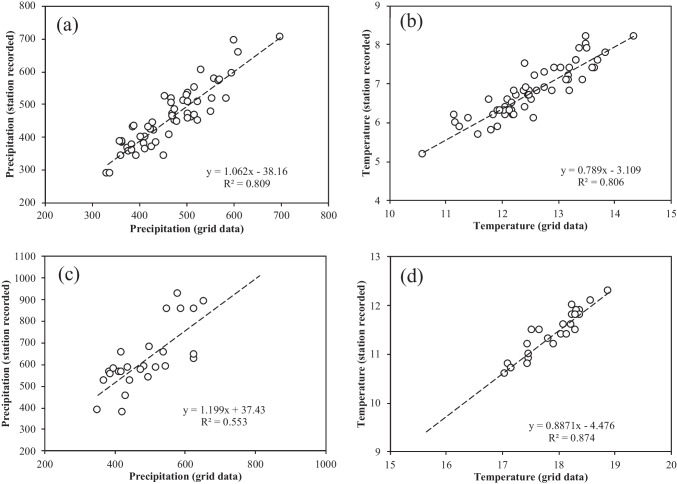
Comparison of station measured precipitation (**a**, **c**) and temperature (**b**, **d**) with records derived from gridded data products at the Fengning (**a**, **b**) and Miyun (**c**, **d**) meteorological stations located inside the Miyun Reservoir Basin

Annual precipitation and temperature at the five sampling sites were determined using the TerraClimate gridded climatic data product (Abatzoglou et al. 2018). TerraClimate provides monthly precipitation and maximum temperature (plus other variables, including evapotranspiration and soil water, etc.) records, which were aggregated to annual profiles for analysis purposes in this study. The data were accessed and processed using the Google Earth Engine coding platform (https://code.earthengine.google.com/). Previous studies have assessed the accuracy of this data product and concluded that the product agreed well with station records at river basin levels (Zhao et al. 2019). To further check the representativeness of the gridded precipitation and temperature in the MRB region, this study collected the long-term records (accumulated precipitation and averaged daily temperature) at Fengning (data available from 1958) and Miyun (data available from 1989) inside the region and compared them with the gridded records at the station locations. The comparisons indicated overall good agreement between the two sources (Fig. 2). Therefore, the precipitation and temperature derived from TerraClimate should relatively well represent the different climate conditions at the five sampling sites across the MRB.

#### Environmental variables

To analyze the potential impacts of regional air pollution on forest development, this study focused on the major pollutants observed in Beijing and surrounding provinces. The pollutants included PM10, PM2.5, dust deposition, N deposition, and S deposition. Data on pollutant concentration levels were extracted from published articles (Chan and Yao 2008). The Institute of Atmospheric Physics, Chinese Academy of Sciences provided the most recent monitoring results (after 2006) to create a relative long-term dataset.

This study estimated pollutant levels in historical periods based on available instrumental data in recent years. For particulate pollutants (PM10, PM2.5, and dust deposition), records are available from 1998, whereas gaseous pollutant (presented as N and S deposition) concentration data are available from 2006. To create a long-term dataset covering the entire study period which generally started in the early 1960s, the study reconstructed historical levels based on environmental Kuznets curve (EKC) theory. EKC theory asserts that environmental quality deteriorates initially and then improves as the economy develops (Dinda 2004). This pattern is due to the initial enormous inputs of the environment for development combined with increased waste production, while as the entities grow, consumption habits and structural changes take place and result in great environmental protection. Many studies have presented cross-sectional evidence on the relationships between different indicators of environmental quality and per capita national income across countries. For pollution studies specifically, an inverse relationship between the levels of air pollutants and per capita gross domestic product (GDP) was reported (Dinda et al. 2000; Dinda 2004).

This study examined the EKC theory between the selected variables and per capita GDP. The GDP series was derived from the statistical yearbook of Beijing. As illustrated in Table 2, all pollutants showed similar significant inverse relationships with per capita GDP. Regression models were derived based on the years with both pollutant records and GDP data. The models were then applied to the extra years to estimate the historical levels.

**Table 2:**
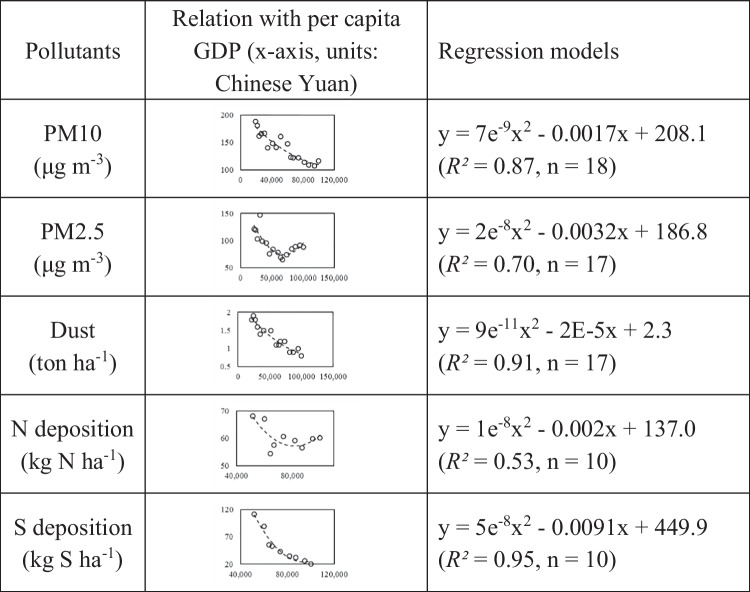
Relationships of major pollutants with the per capita GDP of Beijing

### Data analysis

The temporal profiles of each single dependent (climatic and pollutant variables) and independent variable were compared through one-way ANOVA, using IBM SPSS Statistics 20 (SPSS Inc., USA). The Pearson correlation coefficients between tree ring-derived variables and the dependent variables were calculated to explore possible correlations between the two. Multiple linear regression analysis was used to test the relationship between tree ring-derived and environmental variables as well as the contribution of specific environmental variables.

## Results

### Historical climate conditions and pollution levels

**Fig. 3:**
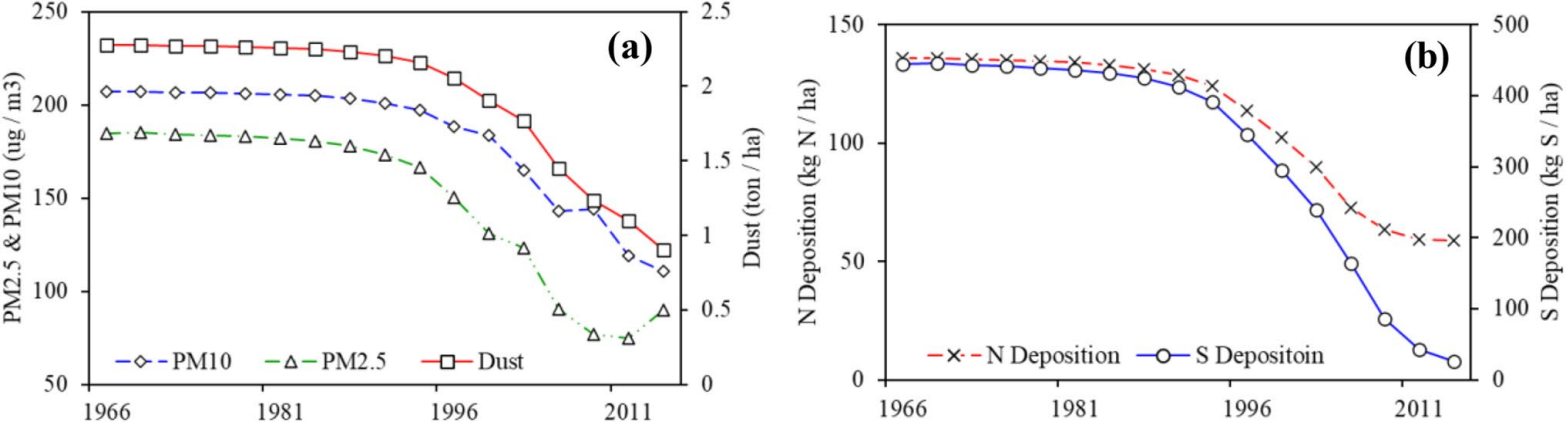
Reconstructed concentration profiles for **a** PM2.5, PM10, and dust deposition; and **b** N and S depositions since 1966

As shown in Fig. 3, the annual average pollutant concentrations were consistently high, at near-constant levels in early years. Significant changes occurred in approximately 1996, when all pollutants presented substantial decreases afterward. Specifically, particulate pollutants (including PM10, PM2.5, and dust depositions) have significantly decreased. N deposition has also decreased from a near-constant level of 132.8 kg N ha^−1^ (averaged value from 1966 to 1993) to approximately 60.4 kg N ha^−1^ (averaged value from 2008 to 2014), and the increasing vehicular population and the resulting high emissions limited its further decrease. S deposition has decreased substantially since the early 1990s to the current low levels, which was mainly due to the relocation of high emission plants and high pollution industries, as well as the adoption of cleaner technologies.

### Overall characteristics of P. tabuliformis growth

**Fig. 4:**
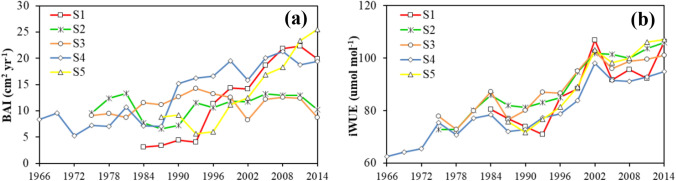
Temporal variation in **a** basal area increment (BAI) and **b** intrinsic water-use efficiency (*iWUE*) at the five selected study sites (S1 to S5)

The BAI showed an overall increasing trend for the five sites, but varied increasing rates were observed (Fig. 4a). S1 and S5 presented the highest growth rates, especially since the middle 1990s. At the initial phase of S1, a low rate of increase of 0.13 cm^2^ year^−1^ was observed, whereas at S5, an initial decrease was observed until 1996. At S4, the BAI was averaged at approximately 7.78 cm^2^ year^−1^ with small interannual variations before 1990, followed by a constant increase (Fig. 4a). S2 and S3 presented similar growth trajectories, and significantly lower growth rates than the other three sites were observed since the late 1990s (Fig. 4a). It is worth noting that the tipping point within the BAI profiles showed some consistency (e.g., mid-1990s) with that for pollutant concentration changes.

The *iWUE* presented similar increases for trees at the five sites (Fig. 4b). Since the *iWUE* is largely influenced by climate change (*c*_*a*_, precipitation and temperature), the synchronized *iWUE* increasing trend might indicate that environmental change, rather than climate change, caused the variations in *P. tabuliformis* growth along the gradient.

**Fig. 5:**
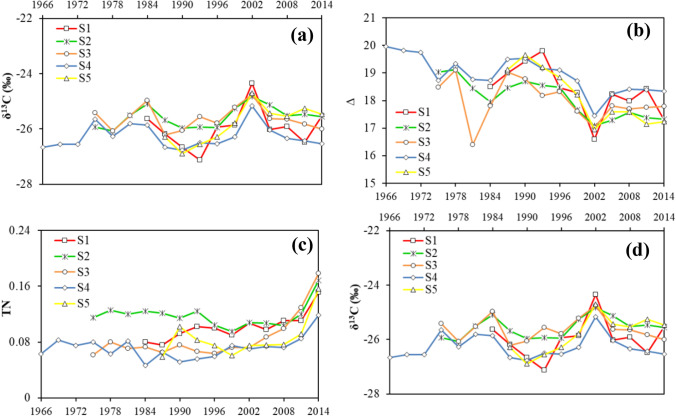
Temporal variations in **a** carbon isotope ratio (δ^13^C), **b** carbon isotope discrimination rate (∆), **c** total nitrogen concentration (%) (TN), and **d** nitrogen isotope ratio (δ^15^N) at the five studied sites

Rising *c*_*a*_ presented overall similar impacts on trees at the five sites as indicated by the trend of the C isotope ratio (Fig. 5a) and the C isotope discrimination rates (Fig. 5b). The synchronized appearance of peaks and valleys reflected the similar mechanisms at the five sites responding to *c*_*a*_, while the differences in the magnitude of ratios might be attributed to extra impacts that suppressed or stimulated the C assimilation procedures.

Total N was maintained at near-constant levels for all sites, but since 2008, abrupt increases were observed. Since N fertilizer application was restricted in this area for the protection of drinking water, the most likely contributor was atmospheric N deposition after the 2008 Olympic Games. The nitrogen isotope ratio (δ^15^N) presented significant variations which might be due to temporal variations in N deposition (lower δ^15^N) and N losses due to denitrification and leaching from heavy rainfall events (with high δ^15^N then) (Sun et al. 2010; Succarie 2020).

### Relationships between tree growth and climatic and environmental changes

Pearson’s correlation analysis was performed among the tree ring-derived variables and climatic and environmental variables. Across the five sampling sites, the tree *iWUE* was found to be significantly correlated to *c*_*a*_ (coefficients ranging from 0.790 to 0.951, Table 3). Temperature (*T*) had positive impacts on *iWUE*, but a significant correlation was observed only at S1 (*r* = 0.606, *p* = 0.048) and S4 (*r* = 0.675, *p* = 0.003). Precipitation (*P*) showed a similar negative control of *iWUE* with varied levels of correlations. All pollutant contents (PM10, PM2.5, dust, N, and S depositions) showed significant negative impacts on the *iWUE* at all sites.

**Table 3:** Pearson’s correlation coefficients between tree ring-derived variables and climatic and environmental variables at the five studied sites. * indicates that the correlation is significant at the 0.05 level (2-tailed); and ** indicates that the correlation is significant at the 0.01 level

Site		*c*_*a*_	*P*	*T*	PM10	PM2.5	D	N	S
S1	*iWUE*	0.790^**^	− 0.422	0.606^*^	− 0.782^**^	− 0.761^**^	− 0.772^**^	− 0.805^**^	− 0.791^**^
	BAI	0.947^**^	− 0.210	0.387	− 0.921^**^	− 0.987^**^	− 0.929^**^	− 0.976^**^	− 0.959^**^
S2	*iWUE*	0.951^**^	− 0.447	0.401	− 0.910^**^	− 0.929^**^	− 0.899^**^	− 0.938^**^	− 0.922^**^
	BAI	0.355	− 0.203	0.079	− 0.426	− 0.517	− 0.425	− 0.487	− 0.456
S3	*iWUE*	0.904^**^	− 0.470	0.474	− 0.845^**^	− 0.886^**^	− 0.836^**^	− 0.891^**^	− 0.871^**^
	BAI	0.203	0.312	− 0.281	0.002	− 0.149	− 0.011	− 0.076	− 0.051
S4	*iWUE*	0.919^**^	− 0.578^*^	0.675^**^	− 0.853^**^	− 0.879^**^	− 0.837^**^	− 0.887^**^	− 0.867^**^
	BAI	0.888^**^	− 0.203	0.533^*^	− 0.782^**^	− 0.864^**^	− 0.786^**^	− 0.845^**^	− 0.820^**^
S5	*iWUE*	0.935^**^	− 0.669^*^	0.379	− 0.927^**^	− 0.928^**^	− 0.912^**^	− 0.952^**^	− 0.938^**^
	BAI	0.935^**^	− 0.590	0.225	− 0.963^**^	− 0.882^**^	− 0.967^**^	− 0.922^**^	− 0.949^**^

For the BAI, there was a significant positive correlation with *c*_*a*_ at S1 (*r* = 0.947, *p* < 0.001), S4 (*r* = 0.888, *p* < 0.001), and S5 (*r* = 0.935, *p* < 0.001), whereas similar positive correlations existed at S2 and S3 at insignificant levels. Significant negative impacts of air pollutants on the BAI were also observed at all five sites except for S2. An insignificant relationship was found between the BAI and the listed climatic variables (Table 3).

**Fig. 6:**
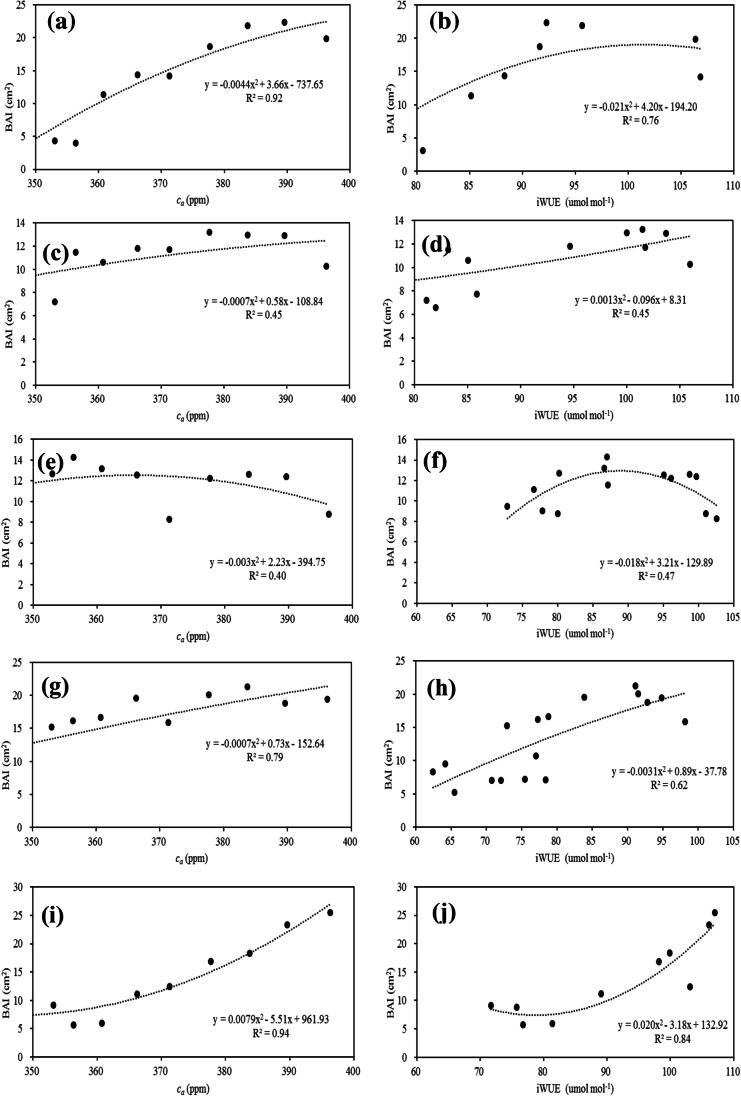
Non-linear correlations between the BAI and *c*_*a*_ (**a**, **c**, **e**, **g**, and **i**), and between the BAI and *iWUE* (**b**, **d**, **f**, **h**, and **j**) at the five selected sites (from S1 to S5)

Figure 6 further indicates a stronger nonlinear relationship between the BAI and *c*_*a*_ and between the BAI and *iWUE*. Overall, the direction of the quadratic relationships between BAI and *c*_*a*_, and the BAI and *iWUE* for each individual site was similar (with varied significance) except that at S5. For S1 and S2, the quadratic functions approached their tipping points, which were calculated to be 415 ppm and 414 ppm *c*_*a*_ levels, respectively. At S3, the maximum BAI was recorded at approximately 371 ppm, and decreasing BAI rates were observed afterward. The BAI at S4 was increasing as well, and it is expected to reach the tipping point when future *c*_*a*_ levels reach approximately 518 ppm. At S5, trees passed a stage with the lowest growth rate when the *c*_*a*_ was approximately 349 ppm, and a constant increasing BAI was observed afterward.

Although the *iWUEs* at the five sites presented similar increasing trends, the BAI responses to the *iWUE* differed between the sites as well. At S1 and S3, the BAI positively responded to the increasing *iWUE* at the initial stages but started to decrease once the *iWUE* reached 101 µmol mol^−1^ and 89 µmol mol^−1^, respectively. S4 presented a similar quadratic relationship between the BAI and *iWUE*, except it had not yet reached the tipping point (approximately 144 µmol mol^−1^) yet. Instead, S2 and S5 presented opposite relationships between the BAI and *iWUE*. Initially, the *iWUE* was likely to restrict tree growth. However, in recent stages, the increased BAI was consistent with the increasing *iWUE*. Typically, with the *iWUE* above the level of 79 µmol mol^−1^, the BAI at S5 showed a linear increase in response to the increased *iWUE*. The similar BAI trajectories but different reactions to the *iWUE* changes might indicate different mechanisms controlling tree growth among the sites.

The multiple regression results (Table 4) indicated the contributions of multiple factors to the BAI changes. A large portion of the variations in the BAI at S1 (91.9%), S2 (45.1%), S3 (40.3%), S4 (79.3%), and S5 (94%) were explained by the elevated *c*_*a*_. At S1 and S5, including additional climatic (temperature, precipitation) and environmental (N deposition, S deposition, dust, PM10, PM2.5) related variables in the models slightly improved the model performance, which indicated that *c*_*a*_ was the controlling factor at these two remote sites with relatively high elevations (Table 1). At S2, temperature and water availability (indicated by the *iWUE*) did not show significant contributions to the BAI, but the dust, N, and S depositions together explained approximately 74.5% of the BAI variations, indicating there were significant effects from air pollutants. At S3, including the *iWUE* and temperature in the BAI-*c*_*a*_ function increased the coefficient of determination by 19%, while the *c*_*a*_, temperature, *iWUE* and pollutants (dust, N and S depositions, PM10, PM2.5) together explained approximately 90% of the BAI variations. The same set of variables (excluding *c*_*a*_) showed less contribution to the BAI changes at S4 after *c*_*a*_ had played a dominant role (explained 79.3% of the BAI variations) at this site.

**Table 4:** Multiple regression analysis for basal area increment (BAI) with climatic (*c*_*a*_, *iWUE*, temperature) and environmental (total N, dust deposition, S deposition, nitrate deposition, PM10, PM2.5) related variables at the five selected sites (S1–S5)

Site	Regression models	Model performance
S1	*BAI* = − 0.0044 × *c*_*a*_^*2*^ + 3.66 × *c*_*a*_—737.65	*r*^*2*^ = 0.919, *p* < 0.001
	*BAI* = − 0.006 × *c*_*a*_^2^ + 4.85 × *c*_*a*_—2.80 × *T* + 0.15 × *iWUE*—932.82	*r*^*2*^ = 0.946, *p* = 0.001
	*BAI* = − 0.002 × *c*_*a*_^2^ + 1.78 × *c*_*a*_—164.34 × TN—404.59	*r*^*2*^ = 0.966, *p* < 0.001
	*BAI* = 0.42 × *c*_*a*_—1.004 × *T* + 0.39 × *iWUE*—0.082 × PM10—0.84 × PM2.5 -14.41 × D + 1.53 × N + 0.012 × S – 169.39	*r*^*2*^ = 0.999, *p* = 0.003
S2	*BAI* = − 0.0007 × *c*_*a*_^2^ + 0.58 × *c*_*a*_—108.84	*r*^*2*^ = 0.451, *p* = 0.067
	*BAI* = − 0.001 × *c*_*a*_^2^ + 0.52 × *c*_*a*_ + 0.069 × *iWUE* + 0.22 × *T* – 99.87	*r*^*2*^ = 0.468, *p* = 0.290
	*BAI* = 0.002 × *c*_*a*_^2^—1.71 × *c *_*a*_- 81.28 × TN + 198.55	*r*^*2*^ = 0.695, *p* = 0.018
	*BAI* = − 0.060 × *c*_*a*_ + 13.76 × D- 0.19 × TN- 0.024 × S + 33.048	*r*^*2*^ = 0.764, *p* = 0.023
	*BAI* = 12.29 × D—0.19 × N- 0.012 × S + 10.66	*r*^*2*^ = 0.745, *p* = 0.009
S3	*BAI* = − 0.003 × *c*_*a*_^2^ + 2.23 × *c*_*a*_—394.75	*r*^*2*^ = 0.403, *p* = 0.058
	*BAI* = − 0.003 × *c*_*a*_^2^ + 2.41 × *c*_*a*_—0.12 × *iWUE*—1.15 × *T*—424.056	*r*^*2*^ = 0.596, *p* = 0.062
	*BAI* = 0.21 × *c*_*a*_ + 0.008 × *iWUE*—0.42 × *T*—0.027 × PM10—0.42 × PM2.5—8.82 × D + 0.83 × N + 0.013 × S—74.015	*r*^*2*^ = 0.879, *p* = 0.056
S4	*BAI* = − 0.0007 × *c*_*a*_^2^ + 0.73 × *c*_*a*_—152.64	*r*^*2*^ = 0.793, *p* < 0.001
	*BAI* = − 0.001 × *c*_*a*_^2^ + 0.88 × *c*_*a*_—0.11 × *iWUE*—0.12 × *T –* 163.68	*r*^*2*^ = 0.801, *p* < 0.001
	*BAI* = 0.25 × *c*_*a*_—0.26 × *iWUE* + 0.23 × *T* + 0.13 × PM10 + 0.33 × PM2.5 + 4.66 × D—0.53 × N + 0.042 × S—49.72	*r*^*2*^ = 0.872, *p* = 0.007
S5	*BAI* = 0.0079 × *c*_*a*_^2^—5.51 × *c*_*a*_ + 961.93	*r*^*2*^ = 0.940, *p* < 0.001
	*BAI* = 0.009 × *c*_*a*_^2^—6.53*c*_*a*_ + 0.14 × *iWUE*—0.32 × *T* + 1160.93	*r*^*2*^ = 0.946, *p* = 0.002
	*BAI* = 0.12 × *c*_*a*_^2^—8.16 × *c*_*a*_ + 0.044 × *iWUE* + 0.47 × *T*—42.064 × TN + 1447.97	*r*^*2*^ = 0.953, *p* = 0.009
	*BAI* = − 1.018 × *c*_*a*_—0.28 × *iWUE* + 5.50 × *T*—1.97 × D + 0.35 × N—0.10 × S—0.44 × PM10—0.12 × PM2.5 + 445.95	*r*^*2*^ = 0.998, *p* = 0.09

*T*, TN, D, N, and S represent the temperature, total N, dust deposition, N deposition, and S deposition, respectively

### Relationships among rainfall extremes, pollution, and tree ring measurements

**Fig. 7:**
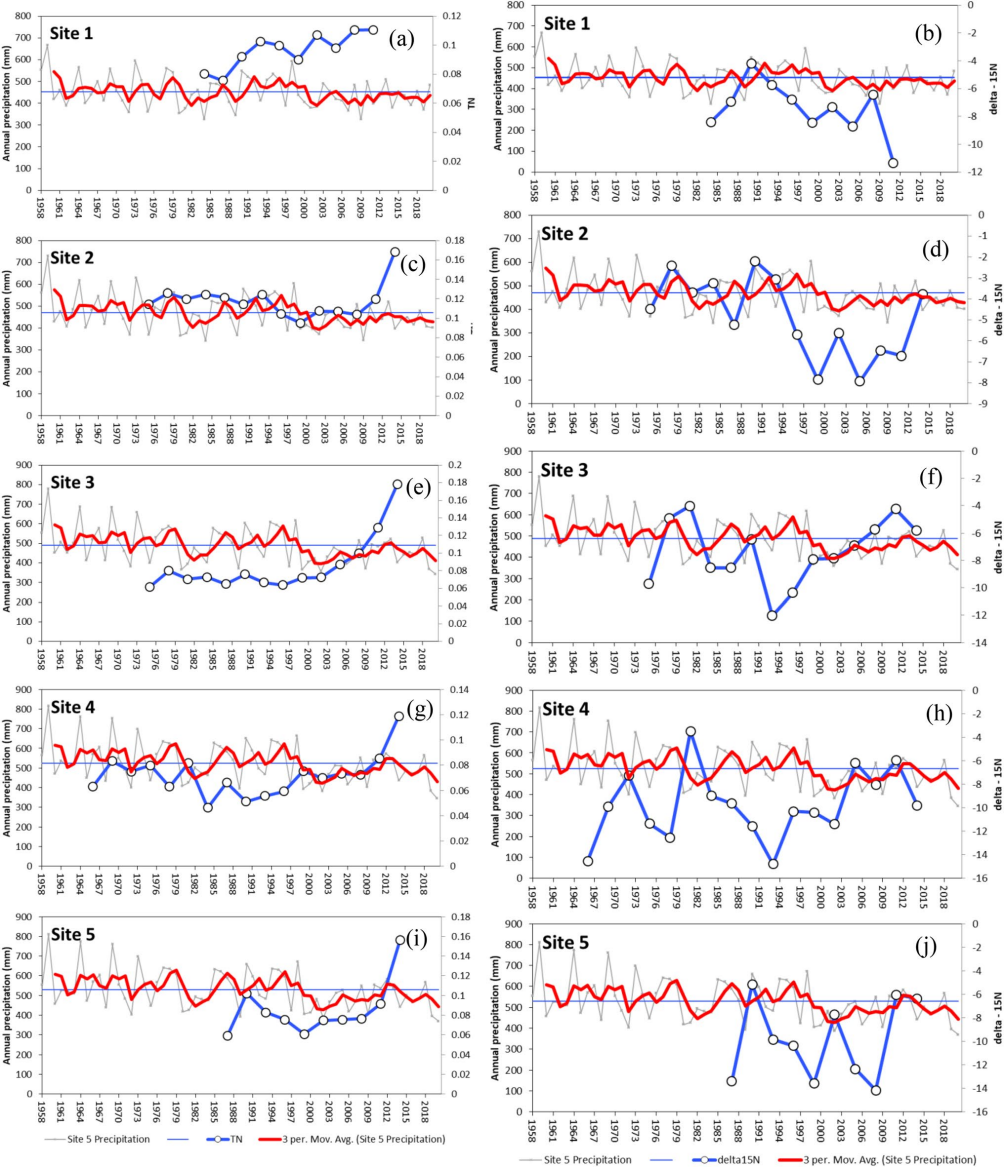
Trends of annual rainfalls, 3-year average rainfall, 3-year average tree total nitrogen (N) concentrations, and 3-year average tree nitrogen isotope composition (δ15N) in *Pinus tabuliformis* in the Miyun Reservoir Basin, Beijing China

**Fig. 8:**
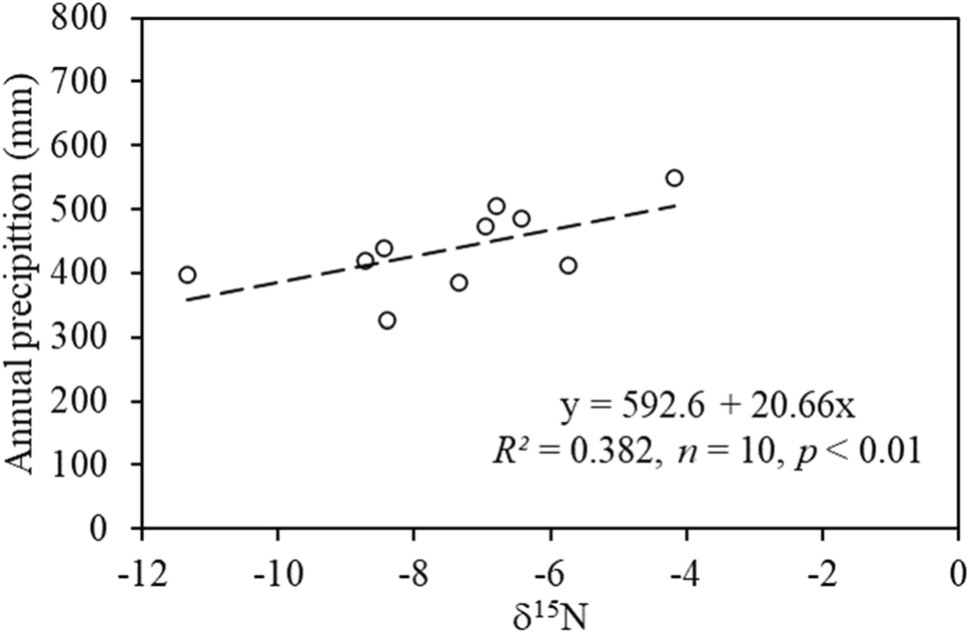
Relationship between mean annual precipitation and mean annual tree ring δ.^15^N at the northern and high-elevation site (S1)

At the two relatively nonpolluted sampling sites (S1 and S2) in the north and high-elevation areas, the tree ring total N seemed to increase (Fig. 7a, c), but the tree ring δ^15^N tended to decrease in the same period (Fig. 7b, d), highlighting that tree N use efficiency appeared to increase with less N losses, as reflected by the general decline in tree ring δ^15^N. It is most interesting but not surprising that the tree ring δ^15^N peaks and valleys in Fig. 7 correspond well with those of rainfall extremes of high and low rainfall. At the other three more polluted sites (S3, S4, and S5), both tree ring total N and δ^15^N seemed to have increased since 1985, highlighting that the N deposition at the sites continued to increase. At the S1 site, which had a high elevation and was a northern site with little N deposition, there was a significant positive relationship between mean annual precipitation and mean annual tree ring δ^15^N, highlighting that a higher annual rainfall would result in a higher N availability as reflected in Fig. 7a, leading to higher N losses and hence a higher tree ring δ^15^N (Fig. 8). Due to the significant N deposition at the other four sites (S2, S3, S4, and S5) (Fig. 7), no such positive linear relationships were detected.

## Discussion

### Annual trends of pollutant concentrations in the MRB

In the present study, the air pollutant concentrations were reconstructed based on the widely used EKC theory (Dinda et al. 2000; Dinda 2004). A relatively high pollution level before the 1980s and a significant decreasing trend since the end of the 1990s were observed. The overall trend of pollutant concentration change can be treated as rational due to the primary reason for the economic structure reforms and the combined technological development since the end of the 1970s. Before the current reforms, economic growth in China largely relied on the consumption of resources, while tons of pollutants were emitted into the atmosphere. Economic transitions after the reforms not only resulted in technological improvement which significantly increased energy efficiency and reduced pollutant emissions but also caused increasing spending on environmental issues in China. Since 1998, dozens of air pollution control measures were implemented in the planning for the 2008 Beijing Olympic Games, as the goal was to decrease pollution (Chan and Yao 2008). Similar decreasing trajectories of pollutants along rising per capita income levels have been previously reported (Kaufmann et al. 1998; Brock and Taylor 2005).

### BAI responses to elevated c_a_ and climate change

Although tree *iWUE* showed a similar increasing trend (Fig. 4), as well as a significantly positive correlation with rising *c*_*a*_, the directions and significance of the relationships between the *iWUE* and BAI differed among the five selected sites. For *P. tabuliformis* at S1, the increasing *iWUE*, as well as its initial positive relationship with the BAI, can be largely attributed to the *c*_*a*_-stimulated CO_2_ assimilation rate. This result agrees well with previous studies that concluded that rising *c*_*a*_ is likely to increase *iWUE* across biomes (Peñuelas et al. 2008; Wang and Feng 2012). However, as the *c*_*a*_ continued to rise, the increasing rate of the annual BAI was reduced, which could be ascribed to increasing water stress-induced stomatal conductance reduction. An overall decrease in precipitation with increasing temperature was observed according to the climate records. The decreased stomatal conductance would help reduce transpiration through leaves and thus maintain the increasing trend in the *iWUE* (Fig. 4). However, reduced stomatal conductance would limit assimilation procedures, which led to the negative relationships between the BAI and *iWUE* for the recent periods. Such a phenomenon (increasing *iWUE* in combination with a quadratic relationship between the *iWUE* and BAI) was also observed at S2, S3, and S4 but with less significance, indicating possible contributions from other variables. However, at S5, the tree growth (BAI) linearly responded to the rising *c*_*a*_, while the BAI and *iWUE* presented an adverse correlation against the other four sites. This result might be ascribed to the relatively abundant water availability at the site; thus, water has not yet limited tree growth. Therefore, the increased *c*_*a*_ led to high photosynthetic activities and thus high *iWUE* levels.

### BAI responses to air pollution

The impacts of air pollutants on tree growth could be inferred from two aspects. First, from a qualitative perspective, only the sites subjected to frequent exposure to high pollutant concentrations showed signs of affecting tree growth, as evidenced by the multiple regression analysis. In the current study, S1 and S5 were recognized as the two sites with relatively less impact from air pollution due to their high elevations and fluctuating terrains (and thus possibly higher humidity and more precipitation, which are not conducive to the distribution of pollutants). Thus, tree growth at these sites showed relatively consistent responses primarily to the increasing *c*_*a*_. Pollutant related variables, together with precipitation and temperature, contributed less than 10% of the BAI variations. However, for S2 and S3, which were located closer to Beijing city with relatively flat terrain characteristics, including air pollutant-related variables in the multiple regression analysis apparently increased the performance of the models. Such a phenomenon could be ascribed to the distance of the sites from the pollution sources, which agreed well with the previous statements that the degree of suppression of tree growth from air pollution would decrease as the distance to the pollution source increased (Sakata and Suzuki 2000; Rydval and Wilson 2012).

From a quantitative perspective, during high-pollution periods in the MRB (e.g., prior to 1996), S2 and S3 presented higher δ^13^C values (and thus lower Δ values). For instance, the δ^13^C in 1984 was recorded to be − 25.10‰ and − 24.97‰ at S2 and S3, respectively, which was approximately 0.5‰ higher than the other sites. In 1993, the δ^13^C at S2 and S3 was − 25.93‰ and − 25.56‰, respectively, while the corresponding value at S1 was − 27.12‰. The difference in δ^13^C among the sites diminished somewhat as less pollution was observed after 1996. This could be explained by the stomatal closure caused by severe air pollution, leading to poor tree growth. Studies also found that trees exposed to multiple air pollutants would result in less negative δ^13^C values, which the authors attributed to stomatal closures as well (Martin et al. 1988; Rinne et al. 2010). The greater determination of coefficient (*r*^*2*^) obtained when including the dust, N, and S deposition in the multiple regression analysis at S2, S3, and S4 also suggested that pollutants played important roles in determining tree growth rates. Typically, at S2, when *c*_*a*_ and other climatic-related variables were removed from the regression model, the contribution of pollutants increased and was greater than that from climatic variables, highlighting that pollutants played a key role in tree growth at this site. However, at S5, the BAI experienced an initial decrease before the *iWUE* come to the level of 79 µmol mol^−1^, which occurred between 1993 and 1996. Considering the heavy pollution for the period, this BAI reduction might also be attributed to the pollution-induced *g*_*s*_ decrease, while the following BAI increase might indicate that *P. tabuliformis* adapted to the pollution and unrestricted (by *g*_*s*_ closure) photosynthetic activities were performed afterward. The negative correlations between the BAI and the dust, N deposition, S deposition, PM10, and PM2.5 at the five selected sites added further evidence of pollution induced stomatal limitation on tree growth.

Although the impacts of pollution on *P. tabuliformis* growth along the gradient have been discussed by analyzing the spatial and temporal characteristics of both tree ring derived growth variables and environmental variables, there are some limitations in this study. First, no spatial distribution of pollutants is available to discuss the detailed impacts of air pollution. The high fluctuations within tree ring δ^15^N values highlighted the sensitivity and usefulness of tree ring δ^15^N for further quantifying the contribution of N, for example, the significantly higher δ^15^N due to heavy N deposition and the higher N losses or higher δ^15^N in tree rings due to the significant N losses caused by denitrification and leaching under heavy rainfall events (Sun et al. 2010; Succarie et al. 2020). The S isotope ratio should also be considered to quantify the impacts of pollution. Finally, only one species was studied, and it is difficult to extend the derived conclusions to the other tree species in the area.

## Data Availability

Please contact the listed corresponding author for accessing the raw data used in the paper.
